# Special Issue “Recent Advances in Molecular and Cellular Research in Ophthalmology”

**DOI:** 10.3390/ijms27136028

**Published:** 2026-07-05

**Authors:** Zala Lužnik Marzidovšek

**Affiliations:** 1 Eye Hospital, University Medical Centre Ljubljana, 1000 Ljubljana, Slovenia; zala.luznik@kclj.si or zalaluznik@gmail.com; 2 Medical Faculty, Department of Ophthalmology, University of Ljubljana, 1000 Ljubljana, Slovenia

Various ocular diseases are a major cause of visual impairment and blindness worldwide [1]. Advances in molecular biology, genetics, and regenerative medicine have significantly improved our understanding of the mechanisms underlying these conditions. The identification of genetic risk factors, biomarkers, and disease-specific molecular pathways is driving the development of more precise diagnostic tools and innovative therapeutic strategies, including gene therapy, stem cell-based approaches, and other regenerative treatments aimed at preserving and restoring vision [2]. 

To highlight these advances, this Special Issue, Recent Advances in Molecular and Cellular Research in Ophthalmology, brings together three original research articles and two review papers that focus on recent progress in molecular genetics, ocular surface biology, regenerative medicine, and gene-based therapies.

- **An Overview of Published Articles**

In their original research article, Amini et al. (Contribution 1) investigated the role of miR-204-5p in regulating gene expression in limbal epithelial cells under both physiological and inflammatory conditions. Limbal epithelial cells are essential for corneal epithelial renewal and ocular surface homeostasis. They demonstrated that miR-204-5p modulates genes involved in cellular differentiation, structural integrity, retinoic acid signaling, and inflammation, with notable effects on FOXC1 and KRT3 expression. Importantly, these proteins were identified as novel targets of miR-204-5p during lipopolysaccharide-induced inflammation. These findings provide new insights into the molecular mechanisms governing limbal epithelial cell homeostasis and suggest a potential role for miR-204-5p in ocular surface inflammation and regeneration.

Next, in their review article, Kobal et al. (Contribution 2) examined the emerging role of mesenchymal stem cells (MSCs) and MSC-derived extracellular vesicles (MSC-EVs) in corneal regeneration. They summarize the molecular and cellular mechanisms underlying the regenerative, anti-inflammatory, and wound-healing effects of these therapies, highlighting their potential for treating conditions such as corneal epithelial defects, dry eye disease, and keratoconus. The review also discusses the first clinical trial findings and highlights the potential of MSCs and MSC-EVs as promising therapies for corneal diseases, while emphasizing the need for further studies before widespread clinical application.

Similarly, in their review article, Anton et al. (Contribution 3) explored the growing potential of gene therapy as a novel approach for the treatment of glaucoma, a leading cause of irreversible vision loss worldwide. The review summarizes current knowledge of glaucoma-associated genes, including *MYOC*, *OPTN*, and *WDR36*, and discusses emerging gene-based strategies aimed at lowering intraocular pressure and protecting retinal ganglion cells from degeneration. The authors highlight advances in genetic therapies that target aqueous humor dynamics and neuroprotection, addressing limitations of conventional treatments. The findings suggest that gene therapy may offer new opportunities for glaucoma treatment, although additional preclinical and clinical studies are required to establish its long-term safety and efficacy.

In the next article, Bjeloš et al. (Contribution 4) present two patients with retinitis pigmentosa associated with pathogenic variants in the *IDH3A* gene, expanding the spectrum of genotype–phenotype correlations linked to this rare inherited retinal disorder. Their findings suggest that the newly reported *IDH3A* c.293C>T, p.(Pro98Leu) variant is associated with a more severe clinical phenotype, whereas the c.364G>A, p.(Ala122Thr) variant may represent a hypomorphic allele that results in milder retinal disease. Importantly, the study demonstrates that macular pseudocoloboma can occur even in the absence of a null variant and provides valuable insights into the clinical consequences of different *IDH3A* mutations.

Finally, Bobreshova et al. (Contribution 5) investigate the genetic basis of Hermansky–Pudlak syndrome (HPS) in a cohort of patients initially diagnosed with albinism. The authors identified both novel and previously reported variants in the *HPS1*, *HPS6*, and *BLOC1S6* genes, expanding the known genetic spectrum of HPS and its associated ocular manifestations. Their findings underscore the importance of comprehensive genetic testing, interdisciplinary evaluation, and advanced diagnostic approaches, including RNA analysis, for accurate diagnosis and appropriate clinical management of this rare and heterogeneous disorder.

In conclusion, the articles collected in this Special Issue highlight the growing impact of molecular and cellular research on our understanding and management of ocular diseases. Together, they demonstrate how advances in genetics, gene therapy, regenerative medicine, and molecular biology are paving the way toward more precise, personalized, and effective treatments. By bridging basic science and clinical practice, these studies contribute to the ongoing development of innovative strategies aimed at preserving and restoring vision. We hope that the contributions presented in this Special Issue will stimulate further research and foster collaboration in the field of ophthalmology.
